# Effect of benzene extract of *Ocimum sanctum* leaves on cauda epididymal spermatozoa of rats 

**Published:** 2011

**Authors:** Mukhtar Ahmed, R. Nazeer Ahamed, Ravindranath H Aladakatti, Mukhtar Ahmed G Ghodesawar

**Affiliations:** 1 Department of Post-Graduate Studies and Research in Zoology, Karnatak University, Dharwad-580003, India.; 2 Central Animal Facility, Indian Institute of Science, Bangalore-560012, India.; 3 Research Centre, Electron Microscope Unit, College of Science, King Saud University, Post Box 2455, Riyadh 11451, Kingdom of Saudi Arabia.; 4 Department of Zoology, Anjuman Arts, Sciences and Commerce College, Bijapur-586101, India.

**Keywords:** *Ocimum sanctum*, *Epididymis*, *Spermatozoa*, *Fertility*, *Electron microscope*, *Rats*

## Abstract

**Background:**

Recent studies have shown that benzene extract of *Ocimum sanctum (O. sanctum) *leaves induces the ultrastructural changes in the epithelial cells of the cauda epididymis, its subsequent recovery in the seminiferous epithelium and fertility of male albino rats.

**Objective:**

Our aim was to investigate the effect of benzene extract of *O.sanctum *leaves on the cauda epididymal sperm parameters, morphology and their organelles at the ultrastructural level in albino rats.

**Materials and Methods:**

Wistar male rats (n=20) were allocated into two groups of control (n=10) and test group (n=10). The test group received benzene extract of *O.sanctum *leaves (250mg/kg/day) for 48 consequence days. Five animals from each group were used for fertility test. Twenty-four hours after the last dose, the rest of the control (n=5) and treated (n=5) animals were sacrificed by cervical dislocation and then the cauda epididymal plasma was used for sperm analysis, scanning electron microscopy (SEM) and transmission electron microscopic (TEM) studies.

**Results:**

Sperm analysis of test group exhibited significant (p≤0.001) decrease in the sperm count, motility, speed and increase in sperm anomalies when compare to control group. SEM and TEM observation in treated animals indicated the morphological changes in plasma membrane as well as in the acrosomal membrane of spermatozoa, formation of a balloon-like cytoplasmic droplet in the mid-region of abnormal tail and disorganization or degeneration of mitochondria of sperm mitochondrial sheaths.

**Conclusion:**

The effects observed in this study may have resulted from a general alteration in the cauda epididymal milieu, probably due to androgen deficiency consequent to the anti-androgenic property of *O.sanctum *leaves.

## Introduction

Fertility control is an issue of global and national public health concern. Through increased public awareness, statements supporting research on male methods and greater involvements of men reproductive health have been forthcoming from several quarters, including international women's organizations. The clinical and scientific basis for the research has been reviewed in recent years. Apart from research for finding harmless chemical drugs as effective oral contraceptives in the western countries, the crude vegetal drugs used by tribal people are being closely looked into for their possible efficiency to find out safe and effective oral drugs for controlling human fertility. Many of the plants which are common in India are reported to possess antifertility activity as spermicidal, abortifacient or antiandrogenic in nature (1-7). Therefore, it has become necessary to use biologically active botanical substances or fertility-regulating agents of plant origin which are ecofriendly in approach and interfere with the natural patterns of reproduction (8).

The plants of genus *Ocimum *belonging to family Labiatae are very important for their unique properties. *Ocimum sanctum L. *(Tulsi), *Ocimum gratissium *(Ram Tulsi), *Ocimum canum *(Dulal Tulsi), *Ocimum basilicum *(Ban Tulsi),* Ocimum kilimandscharicum, Ocimum ammericanum, Ocimum camphora *and *Ocimum micranthum *are examples of known important species of genus *Ocimum* which grow in different parts of the world and are known to have medicinal properties (9-11). *Ocimum sanctum* is, a small herb seen throughout India, commonly cultivated in gardens. In traditional systems of medicine, different parts (leaves, stem, flower, root, seeds and even whole plant) of *Ocimum sanctum*, have been recommended for the treatment of bronchitis, bronchial asthma, malaria, diarrhea, dysentery, skin diseases, arthritis, painful eye diseases, chronic fever, insect bite etc. The *Ocimum sanctum* L. has also been suggested to possess antifertility, anticancer, antidiabetic, antifungal, antimicrobial, hepatoprotective, cardioprotective, antiemetic, antispasmodic, analgesic, adaptogenic and diaphoretic actions. 

In addition, the leaves of *O. sanctum* significantly altered the sperm count, motility, velocity and fructose contained in the cauda epididymis (12), reduce the mating behaviour of both male and female albino rats (13-15). Recent studies shown that benzene extract of *Ocimum sanctum *leaves induces the ultrastructural changes in the epithelial cells of the cauda epididymis, its subsequent recovery, after withdrawal of treatment, in the process of spermatogenesis and fertility of male albino rats (16,17) and morphological changes in the rat cauda epididymal sperms upon graded dose treatment (18). 

As there is little information concerning the influence of *O. sanctum* leaves on the cauda epididymal sperm at the ultrastructural level, the present investigation is designed to study whether benzene extract of *O.sanctum *leaves could cause some of the sperm parameters, morphological alterations in cauda epididymal spermatozoa and its organelles by electron microscopic studies and fertility of male of albino rats as this medicinal plant has anti-spermatogenic and anti-androgenic like properties (12-15). 

## Materials and methods

**Preparation of test material**

Fresh *O.sanctum* leaves were collected and dried in shade. A voucher specimen (Zoo/herb/File No.47-Acc.No.22) was deposited at Zoology Department, Karnatak University, Dharwad, India. The dried leaves were coarsely powdered and subjected to soxheltation process to get the benzene extract. Extract thus obtained was allowed to dry and stored in a dessicator at 4ºC. The benzene extract is then mixed with propylene glycol as required and administered orally (gavage) to the experimental animals (19).

**Experimental Animals**

Colony bred healthy adult male albino rats (Wistar strain) weighing 190-200g were utilized for experiments. All animals were proven fertility and obtained from the rat colony maintained in the department. They were housed at a temperature of 262^o^C and exposed to 13-14 h of daylight and maintained on a standard rat pellet diet (Gold Mohar, Hindustan Level Ltd., Hyderabad) and water was given *ad libitum*. The animals were acclimatized to the laboratory conditions before conducting experiments and the care of the laboratory animals was taken as per the Committee for the Purpose of Control and Supervision on Experiments on Animals (CPCSEA) regulations. 

**Study protocol**

The control group (n=10) were administered 1 ml propylene glycol/rat/day orally for 48 days and test group (n=10), that received benzene extract of *Ocimum sanctum* leaves (250mg/kg/day) orally for 48 consequence days. The effective dose of 250mg/kg body weight has been arrived at after preliminary studies on dose and duration of 48 days is concerned to the spermatogenic cycle of rat in response studies in our laboratory and reported (12). 

Five animals from each group were used for fertility test. Twenty-four hours after the last dose, rest of the control (n=5) and treated (n=5) animals were sacrificed by cervical dislocation and then the cauda epididymal plasma was used for sperm analysis, scanning and transmission electron microscopic studies. 

**Sperm analysis**

The cauda epididymis was chopped into phosphate buffered glucose saline (PBGS) [composition: NaCl 50 mM/l; Na_2_HPO_4_ 200 mM/l; glucose 200 mM/l and KH_2_PO_4_ 26 mM/l]. The debris was removed and a clear suspension, the epididymal plasma was used for the analysis of total sperm count, sperm motility, forward velocity and relative percentage of abnormal sperms in male albino rats. The total sperm count and motility were calculated according to the method of Besley *et al* (20) using Neubauer’s haemocytometer. Briefly, to increase the accuracy of sperm count, the epididymal plasma was diluted with a spermicidal solution, prepared by dissolving 5 g of sodium bicarbonate (NaHCO_3_) and 1 ml of 40% formaldehyde in 100 ml of normal saline. 

A twenty times dilution was made using W.B.C pipette, which was thoroughly mixed and one drop was added to both sides of Neubauer haemocytometer. The spermatozoa were allowed to settle down in the haemocytometer by keeping them in a humid chamber for one hour. The sperm count was done in R.B.C counting 5 major squares. The total number of sperms were counted in all the major squares and calculated as follows.

Total number of sperms =Total number of sperms per square (x)mTotal volume per square(10-4)×dilution factor (20)

Similarly the total number of motile sperms was calculated, using phosphate buffer saline instead of spermicidal solution. The forward velocity of the sperm was calculated according to the method of Ratnasoorya (21). Briefly, the epididymal plasma was suspended in phosphate buffer saline, cleared the tissue debris and a clear solution was used for the assessment of average forward velocity of sperms. The assessment was made under light microscope, fitted with a movable mechanical stage and a calibrated ocular micrometer, at 400 X magnification. A drop of sperm suspension was transferred to a clean glass slide and the initial place and time of each sperm was recorded. The time taken for forward movement of sperm from the initial place within microscopic field was recorded using a stop watch. The procedure was repeated for 10 spermatozoa in each sample and the average forward velocity of sperm was calculated and expressed as m/sec. The relative proportion of abnormal sperms was analyzed according to the method of Bauer *et al* (22). Briefly, equal volume of cauda epididymal plasma and 5% NaHCO_3_ were taken in a centrifuge tube, mixed well and centrifuged for 5 minutes at 4000g. The supernatant was discarded and 5 ml of normal saline was added to the precipitate, mixed well and centrifuged again. The procedure was repeated 2 to 3 times and a clear precipitate was obtained. To the final precipitate few drops of normal saline were added, mixed thoroughly and a smear was prepared on a clean slide. The smear was dried at room temperature, fixed by heating it over the flame for two to three seconds. Then the smear was flushed with 95% alcohol, drained and dried. It was stained in Ziehl Neelson's Carbol Fuchsin diluted with equal volume of 95% alcohol for 3 minutes and counter stained with 1:3 (v/v) aqueous solution of Loeffer's methylene blue for 2 minutes. After staining, the smear was rinsed in water and dried in air. The abnormal sperms included categories like double tailed, detached head, detached tail, mid piece bending and irregular head. The relative proportion of the normal and abnormal sperms was from the smear and expressed in terms of percentage.

**Preparation of spermatozoa for SEM study**

Preparation of rat spermatozoa for SEM studies was performed as described elsewhere (23). Briefly, a drop of cauda epididymal plasma was fixed in 2% glutaraldehyde, centrifuged and washed with 0.1 M Sodium cacodylate buffer (pH=7.2), centrifuged again in distilled water till the buffer solution was washed out and a thin film was applied on a cover slip, dried, sputter coated with gold and finally observed under scanning electron microscope (Model. LEO 435 VP Detector SL.1. LEO Electron Microscopy ltd Cambridge, England).

**Preparation of cauda epididymal spermatozoa for TEM study**

Preparation of rat spermatozoa for TEM studies was performed as described elsewhere (3). Briefly, the epididymis was removed, rapidly fixed in 3% glutaraldehyde in phosphate buffer (pH=7.4; 0.1 M) for 4 hr at 4°C, washed in phosphate buffer and post-fixed in 1% osmium tetraoxide in phosphate buffer (pH=7.4; 0.1M) for 6 hr. The fixed epididymis was washed several times in distilled water, stained *en bloc *in 2% aqueous uranyl acetate for 6 hr, dehydrated in acetone series, infiltered in epon-araldite mixture for 10 hr and embedded in the same media in a beam capsule. The blocks were cut in LKB Bromma ultramicrotome. Semithin sections of 1 µm thickness were stained with toludine blue for identification of stages. Ultrathin sections were cut at 100-300 A, mounted on copper grids and stained with 1% aqueous uranyl acetate and lead citrate (24). The stained sections were scanned in Jeol-TEM 100 C X II electron microscope for ultrastructural observations. 

**Fertility test**

To assess the fertility rate with reference to the number of implantations, the female rats of proven fertility exhibiting regular estrous or early proestrus stage were separately housed with the control and treated males overnight. The appearance of sperm in the vaginal smear next morning confirmed the mating and is considered as day1 of the pregnancy. After 8 days, the females were laparotomized and the numbers of implantations and pups were recorded. 

**Statistical analysis**

The data were statistically analysed and expressed as Mean± Standard error (25). The comparison of data for statistically significant differences was done using student's t-test and a probability level of p≤0.001 was considered as significant.

## Results

**Sperm analysis**

Analysis of sperm parameters, such as total sperm count, total number of motile sperm, forward velocity of the sperm and percentage of abnormal sperm of the cauda of epididymal plasma were carried out in the control and all the treated animals. The control rats showed 56.40×10^4 ^ total numbers of sperm/ml epididymal fluid, 52.40×10^4^ numbers of motile sperm/ml epididymal fluid with a speed of 127.63 m/sec and 11.40% of abnormal sperm were recorded. Whereas in the 250 mg/kg body weight of benzene extract treated animals showed a highly significant decrease (p≤0.001) in total sperm count (56%), total number of motile sperm (45%), forward velocity of sperm (49%) and a highly significant increase (p≤0.001) in the percentage of abnormal sperm (544%) when compared to control animals (Table I).

**Scanning electron microscopic (SEM) observations of rat sperms from cauda epididymal plasma**

**Figure 1:**
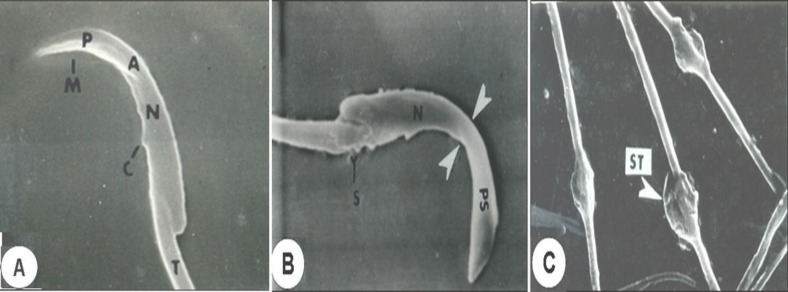
Scanning electron micrographs (SEM) of cauda epididymal spermatozoa of control (Fig.A) and treated with 250mg/kg body weight of benzene extract rat (Figs. B&C)

SEM observations of the cauda epididymal sperms of control rats showed normal parts (Figure 1A). Perforatorium and acrosome are covered with the plasma membrane. A distinguish acrosome is covered with acrosomal membrane. The whole spermatozoon is intact with all the membranes and organelles. However, in 250 mg/kg body weight of benzene extract, treated animals showed disturbance in the plasma membrane as well as in the acrosomal membrane in the most of sperm heads. It is rather difficult to differentiate the acrosomal membrane as well as the plasma membrane. Serrations in the head region of the spermatozoa are observed. The shape and size of the sperm head has also changed considerably. 

There was acute dorsoventrally constriction in the mid-head region of most sperms. The perforatorium (Sub acrosomal material) is bulged/swelled (Figure 1B). Most of the spermatozoa showed a splitting of the tail and distinct visibility of balloon-like cytoplasmic droplets in the mid-region of the tail (Figure 1C).

**Transmission electron microscopic (TEM) observations of Cauda epididymal spermatozoa**

**Figure 2:**
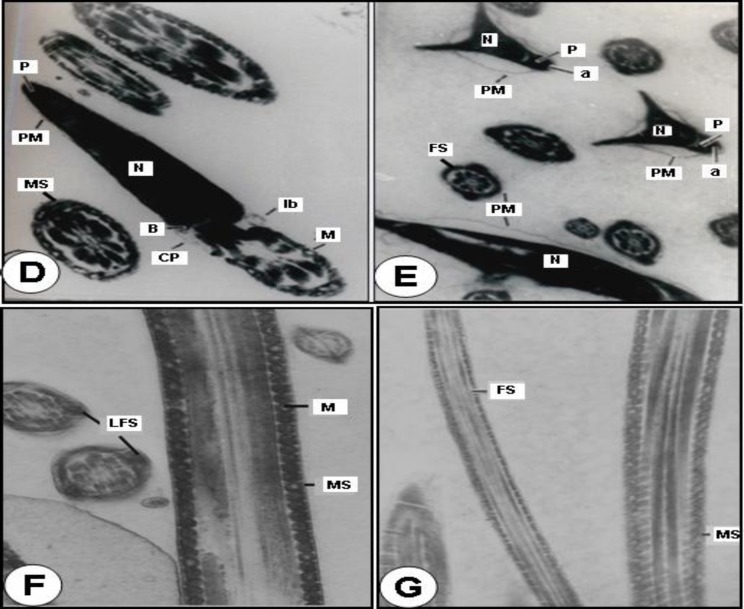
Transmission electron micrographs (TEM) of the control (D-G).

Observations with the TEM revealed that different parts of the cauda epididymal sperm of control rats exhibited normal features (Figure 2D-G).Where as in the animals treated with 250mg/kg body weight of benzene leaf extract, the sperm heads exhibited disrupted plasma membrane, acrosomal membrane and surface coating with fuzzy material. The tip of the sperm head showed disruption of the plasma membrane and acrosome.

**Figure 3:**
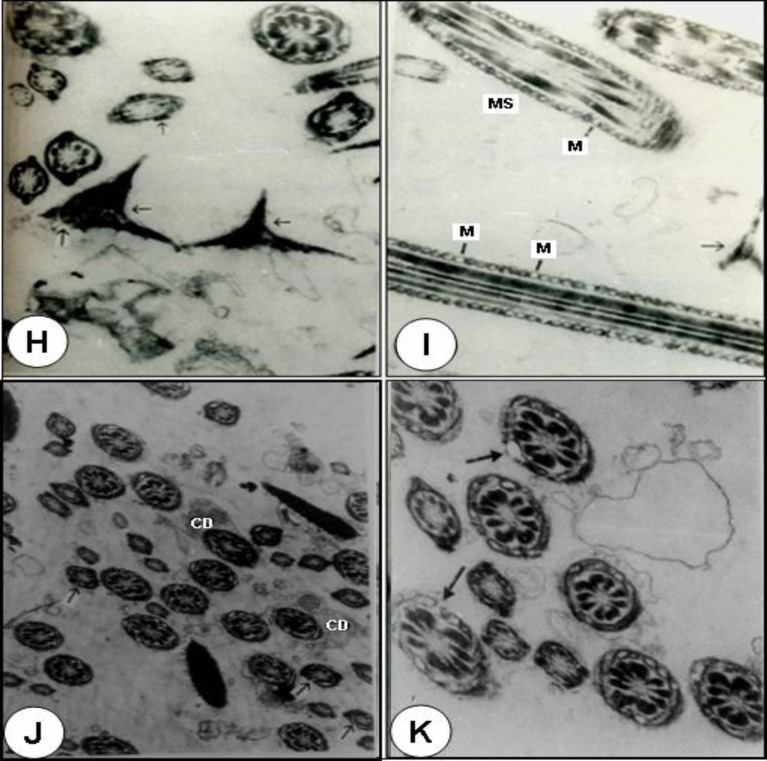
Treated with 250mg/kg body weight of benzene extract rat (Figs. H-K).

The perforatorium was condensed and most of its surface was covered by a thin portion of acrosomal sac. The anterior and caudal portion of the sperm heads revealed disruption or loss of plasma membrane, acrosome, perforatorium and small vesicles on the ventral surface of the perforatorium, which is probably part of the acrosomal system. Most of caudal portion of the sperm head revealed the disturbance of lamellar body and centrioles. The basal plate, posterior ring and post-nuclear cap appeared normal (Figure 3H and I). 

The middle part showed disruption and degeneration of the mitochondrial sheath and a loss of plasma membrane. The disorganization, or commencement of degeneration of the mitochondria, was observed in most of the abnormal mitochondrial sheaths. Some showed a displaced mitochondrial sheath on one or both sides and there was abnormal pattern of outer dense fibres. Different parts of the principle part of the tail exhibited loss and discontinuation of plasma membrane and fibrous sheath, respectively (Figure 3J-L). Most of the tail sections showed retention of cytoplasmic droplets around the middle part on one side (Figure 3K).

**Fertility test**

Results of fertility performance test showed that female rats mated with control male rats illustrated that the numbers of implantations were 10.20±1.07 on day 8 of pregnancy and number of pups obtained 9.60±1.08. However, no implantations were observed in the female rats mated with benzene extract* O. sanctum* leaves treated male rats.

**Table I:** Effect of *O.sanctum* leaves (benzene extract) on various sperm parameters of cauda epididymal plasma in albino rats (values are expressed as SEM of 5 animals).

**Group**	**Treatment**	**Sperm count** **(Total no. x 10** ^4^ **/ml)**	**Motile sperm** **(Total no. x 10** ^4^ **/ml)**	**Forward velocity** **(µm/sec)**	**Abnormal sperms (%)**
I	1 ml propylene glycol	56.40 ± 1.39 (100%)	52.40 ± 2.15 (100%)	127.63 ± 2.75 (100%)	11.40 ± 0.26 (100%)
II	250 mg/kg body weight of benzene extract *O.sanctum* leaves	32.00 ±1.22*** (56%)	24.00 ±1.70*** (45%)	63.16 ±1.71*** (49%)	62.03 ± 1.95*** (544%)

*** p ≤ 0.001

**A.** Spermatozoa of control rat exhibiting normal parts of acrosome (A); post nuclear cap (C); plasma membrane (M); nucleus (N) perforatium (P) and tail region (T). 4.56kx.

**B.** The perforatium (sub-acrosomal material), swells (PS) and the middle region of the sperm head is constricted dorsoventrally (arrows). There is serration (S) at the connective piece of the spermatozoa. A5.56kx.

**C.** There is increase of swelling at tail region in the mid portion of the tail region rat spermatozoa (arrows, ST). 5.93 kx.

**D:** A median section through the base of the sperm head illustrating nucleus (N) covered with perforatorium (P), basal plate (B), plasma membrane (PM) and connecting piece of tail (CP). Caudal portion of the nucleus illustrating lamellar body (lb) and mitochondria (M) are seen, in oblique section, in contact with connecting piece. Cross sections(CS) of mid-piece of spermatozoa flagellum with spatial arrangement of the 9 outer dense fibres to one another, to the centrally located axoneme composed of the 9 plus 2 arrangements of microtubules and to the mitochondrial sheath (MS) X 29,000.

**E.** C.S. of anterior portion of sperm head illustrate normal features of nucleus (N); perforatorium (P); acrosome (a); plasma membrane (PM) and principal piece are with normal features of fibrous sheath (FS) and arrangement of outer fibres and axonemal component X 21,700.

**F-G:** Longitudinal section (LS) of mid piece and principal piece illustrating well-preserved mitochondrial sheath (MS) and fibrous sheath (FS) and are intact with plasma membrane. Mitochondria (M) appear normal. C.S. of principal piece is with normal features of longitudinal fibrous sheath (LFS) and arrangement of outer fibres and axonemal component X 15,000.

**H:** C.S. of the different parts of sperm head (←) showing disruption in normal features plasma membrane, acrosome and perforatorium. Fuzzy material is seen on their surface. X 15,000.

**I:** L.S and C.S. of the mid piece shows the loss of plasma membrane. Most of mitochondria (M) are hypertrophied or have started degeneration. The loss of plasma membrane is seen in the sperm head region (←**) **X 28,000.

**J: **C.S. of the mid piece showing abnormal pattern of mitochondrial sheath and loss of plasma membrane. Most of mid-piece show increased cytoplasmic droplets (CD) displaced on one side are clearly seen. Coating of fuzzy material is seen on the surface of head and tail sections X 10,300.

**K: **C.S of the mid piece shows abnormal pattern and degeneration of mitochondrial sheath (long arrows) along the length of the structure and displaced mitochondrial sheath on one side. The discontinuation of fibrous sheath is also noticed in principal piece X 17,500.

## Discussion

In the present study of benzene extract of *O. sanctum* leaves, treated animals exhibits significant increase in sperm anomalies with reducing sperm count, motility and sperm speed. The epididymis plays an active role in sperm development, and sperm maturation is dependent on the unique luminal environment of the epididymis, including specific proteins synthesized and secreted by the epididymal epithelium (26). Although several epididymis-specific secretory proteins have been identified, little is known about the sperm maturation events in the epididymis (27).

Various plants like methanol sub-fraction of the seeds of *Carica papaya *(4); methanol extract of *Dendrophthoe falcate *(5); ethanolic leaf extract of *Aegle marmelos* (6); hydroalcoholic extract from *Lantana camara* leaves (28); aqueous extracts of *Ruta graveolens L.* solution (29); aqueous extract of *Peganum harmala* (30); and marjoram volatile oil and grape seed extract of *Vitis vinifera* (31) have been reported to possess antifertility activity by reducing above said sperm parameters in mice and rats to support our results. It was suggested that the extract causes androgen depletion at the target level, particularly in the cauda epididymis thereby affecting physiological maturation of the sperm (32). Present observations of increased abnormal sperms, reduced sperm count, motility and sperm speed on treatment with benzene extract of *O. sanctum* leaves, it can be suggested that sperm anomalies in albino rats may have resulted from the alteration in the epididymal milieu due to androgen deficiency following antiandrogenic property of the leaf extract.

**Ultra study of cauda epididymal spermatozoa (SEM and TEM)**

Androgens are essential for survival and motility of spermatozoa in the rat epididymis. Sperm motility is an important attribute of sperm quality as there is a good correlation between sperm motility on one hand and plasma membrane integrity and conception rates on the other. There is an evidence of acrosomal loss or damage as well as other abnormalities observed in caudal region of rat sperm due to this treatment suggesting that these sperms were probably unable to fertilize the ovum (33). From the literature of medicinal plants, it has been shown that different parts of the plant source affecting aspects of male reproduction, brings about the effect through either of two mechanisms namely, estrogenic or antiandrogenic effect and extensively established that these plant sources cause impairing sperm parameters results exhibit androgen is essential for the maturation, motility and survival of sperms in the epididymis (1, 2, 32, 34). 

The acrosome contains several enzymes which are secreted by the Golgi apparatus and endoplasmic reticulum. The production of enzymes destined for the acrosome is regulated to some degree by testosterone (35). From the histochemical evidence, the presence of carbohydrates or polysaccharides in the head of the spermatozoa, which are associated with various enzymatic activities, is indicated. Earlier studies have been reported that morphological changes in the head of spermatozoa in general and the acrosome in particular may have resulted from an alteration in the epididymal milieu of rats treated with crude leaf extract of *Azadirachta indica* (23) and alcohol seed extract of *Momordica charantia* (2) suggested that these changes are due to a general disturbance of carbohydrates or polysaccharides present in the acrosome of the sperm head (23). In the SEM, observation of present study revealed that most of the sperms shown deformity includes dorsoventrally constricted with the bulged sub acrosomal material in the mid region of sperm heads and disrupted plasma membrane and acrosomal membrane particularly at the anterior region on treatment with benzene extract of *O. sanctum* leaves are probably due to the general disturbance in the proteins. 

Specialized structural features of the spermatozoa are a reflection of its unique functional activities. Various authors have suggested that in spite of morphological variations, the main structures present in the sperm head of mammals are the nucleus, the acrosome or acrosomal cap and the membranous envelops (36). The acrosome is unique organelle (37) that is required for fertilization in mammals. It has been predicted that the post-nuclear cap plays a protective role in maintaining the head shape and this structure is very resistant to any extractive agent. The mitochondrial sheath is believed to be the source of energy for sperm motility and outer dense fibres might be contractile because of their close association with the axoneme and play an active role in flagellar motion (37). These outer dense fibres provide added strength to protect sperm from damage by shear forces encountered during epididymal transit or ejaculation (38). Reports on different plants extract namely gossypol, *Solanum xanthocarpum, Carica papaya* cause the sperm abnormalities by exhibiting acrosomal damage and mid-piece anomalies which results in complete inhibition of fertility in rats and mice (32, 39). Crude extract of *Echeveria gibbiflora *on guinea pig sperm, results in the formation of a huge bubble by distension of the plasma membrane and dispersion of the acrosome content with disappearance of the external acrosomal membrane at the sperm head level (40). Triptolide isolated from *Tripterygium wilfordii *cause all cauda epididymal sperm to exhibit a complete absence of the plasma membrane over the entire middle and principal piece and prematured decondensation of the nuclei in rats (41). Similarly, crude extract of *A.indica* leaves (3); aqueous decoction of *Chenopodium album* seeds (7); chloroform extract of *Carica papaya *seeds (42); and a hydroalcoholic extract of *Lantana camara *leaves (28) were shown morphological abnormalities in the head of spermatozoa along with mid-piece anomalies in rats and rabbits. 

The spermatozoa of the cauda epididymis in the present study of benzene extract of *O. sanctum* leaves treated animals revealed several abnormalities. Abnormal patterns of the outer dense fibres and components of axoneme are displaced on one side or both sides, complete absence of plasma membrane of the entire middle and principal pieces and disorganization of mitochondrial sheath in several spermatozoa of rats were observed. The missing segment of the mitochondrial sheath probably represents a weak point in the structural collar, which supports the axial fibre-bundle during the contractional wave, leading to splitting of the axial bundle and subsequent dislocation of its fibres (37, 38). Recent ultra study of aflatoxin B1, a food borne mycotoxin, has shown that the sections of the testis and cauda epididymidis revealed sperm flagella missing one or more outer dense fibres and the associated axonemal microtubule doublets. Severe mitochondrial pathologies in spermatozoa and elongated spermatids, suggesting a link between AFB1-induced sperm mitochondrial pathology due to possibility that AFB1 treatment would disrupt the cytoskeletal proteins of the flagellum in male albino rats (43). It has been suggested that the extract might cause an androgen deprived effect to target organs resulting in alterations in the internal milieu, especially of cauda epididymis (32). 

From both SEM and TEM observations of treated animals showed that the tail portion has balloon like cytoplasmic droplets. Ejaculates containing a high proportion of spermatozoa with attached cytoplasmic droplet can be correlated with altered epididymal function and reduced fertility (44, 45). The presence of high proportion of spermatozoa with attached cytoplasmic droplets in benzene extract of *O. sanctum* leaves treated animals may be due to altered epididymal function. Similar observations were made in studies of combination of progestagen and androgen, *Carica papaya,* vincristine and aflatoxin B1 treated animals (1, 32, 33, 46). Agnes and Akbarsha (46) showed in their experimental study a higher percentage of cauda epididymal spermatozoa retained the cytoplasmic droplets in albino mouse. Cytoplasmic droplets contained electron-dense spherical inclusions, which were hypothesized as lipid inclusions produced from the lamellae through the spherical vesicles of the cytoplasmic droplets.

 It is known that spermatozoa carrying cytoplasmic droplets would be inhibited in motility and may not fertilize the ova (47). Hence, in the present study, in the light of the pathological changes observed, it is suggested that benzene extract of *O.sanctum* leaves damages the spermatozoa in the epididymis, leading to reduced fertilizing ability of the sperm. The fertility studies reveal that the male rats treated with *O.sanctum* leaves are unable to fertilize the female rats probably because the male gametes are affected thereby, establishing the antifertility property of the plant studied. 

## Conclusion

It was demonstrated that the administration of benzene extract of *O. sanctum* leaves can induce the morphological changes in the head and tail region of albino rat sperms.This benzene extract of *O. sanctum* leaves was may be due to general disturbance of proteins and alteration in the epididymal milieu probably due to androgen deficiency consequent upon the antiandrogenic property of this plant. However, these conclusions are based on the preliminary study, where the rats are force fed with the benzene extract of *O. sanctum* leaves. More refined and sophisticated study is needed for identification of active principles and their effects on androgen dependent parts of the spermatozoa in albino rats which are under progress. 
